# Pepsin Triggers Neutrophil Migration Across Acid Damaged Lung Epithelium

**DOI:** 10.1038/s41598-019-50360-4

**Published:** 2019-09-24

**Authors:** Bryan P. Hurley, Rebecca H. Jugo, Ryan F. Snow, Tina L. Samuels, Lael M. Yonker, Hongmei Mou, Nikki Johnston, Rachel Rosen

**Affiliations:** 1 Department of Pediatrics, Mucosal Immunology & Biology Research Center, Massachusetts General Hospital, Harvard Medical School, Boston, Massachusetts USA; 2 Aerodigestive Center, Department of Gastroenterology and Nutrition, Boston’s Children’s Hospital, Harvard Medical School, Boston, Massachusetts USA; 3 Departments of Otolaryngology and Communication Sciences, and Microbiology and Immunology, Medical College of Wisconsin, Milwaukee, Wisconsin USA

**Keywords:** Inflammation, Respiratory tract diseases, Gastroenterology

## Abstract

Pepsin represents a potential biomarker for extraesophageal reflux disease when detected in airways, however a direct role for pepsin in lung dysfunction has not been clearly established. Children experiencing gastroesophageal and extraesophageal reflux are often prescribed proton pump inhibitors (PPIs) to reduce gastric acid associated damage to esophageal and airway mucosa. The potential of pepsin and gastric fluid, from children that were either on or off PPI therapy, to cause inflammation and damage using a human *in vitro* co-culture model of the airway mucosa was evaluated herein. Exposure of the airway model to acidic solutions caused cellular damage and loss of viability, however, acid alone did not disrupt barrier integrity or instigate neutrophil trans-epithelial migration without pepsin. Gastric fluid from patients on PPI therapy exhibited only a slightly higher pH yet had significantly higher concentrations of pepsin and elicited more barrier disruption and neutrophil trans-epithelial migration compared to gastric fluid from patients off PPIs. Inflammatory and damaging responses observed with gastric fluid from patients on PPIs were largely driven by pepsin. These results indicate the potential for PPI usage to raise concentrations of pepsin in gastric fluid, which may enhance the pathological impact of micro-aspirations in children with extraesophageal reflux.

## Introduction

Gastroesophageal reflux (GER) has been implicated as an exacerbating factor for lung disease in children^1^. Understanding the relationship between GER and airway dysfunction has been a challenge without sensitive biomarkers to effectively diagnose extraesophageal reflux disease. One of the proposed biomarkers is the proteolytic digestive enzyme pepsin^1,2^. Pepsin is produced in the stomach where it is autocatalytically cleaved from its proenzyme, pepsinogen. Since pepsinogen is uniquely released and processed to pepsin in the acidic environment of the stomach, the presence of pepsin in the lung suggests transfer of gastric contents to the airway, a consequence of reflux and micro-aspiration. Pediatric studies suggest that in patients with respiratory disease, up to 44% of patients can have detectable pepsin in bronchoalveolar lavage fluid or saliva suggesting an association between pepsin and extraesophageal symptoms^3,4^. Whether pepsin is simply a biomarker or if it plays a role in the pathophysiology of lung disease in children is not known. Pepsin, in the presence of acid, can cause the denuding of laryngeal epithelium with subsequent membrane leakiness as well as elicit the production of inflammatory cytokines^5^. Pepsin has also been shown, even in nonacidic environments, to promote macrophage differentiation and migration as well as induce inflammatory cytokines in tonsillar tissue^6^.

There is significant debate over the role of acid suppression in preventing pepsin-related damage to mucosal surfaces^5,7,8^. Since pepsin is maximally activated at low pH, there is a theoretical benefit to raising the gastric pH using proton pump inhibitors (PPIs) in order to reduce acid related damage; however, PPI treatment does not mitigate reflux and micro-aspiration, it just changes the pH of the refluxate. Furthermore, it has been demonstrated that gastric fluid collected from adult patients receiving PPIs and exposed to airway epithelial cells stimulated a greater inflammatory response compared to gastric fluid from patients not taking PPIs^7^. Specifically, gastric fluid from PPI-treated patients triggered a more substantial release of the chemo-attractant IL-8, which is associated with neutrophil recruitment to the airway mucosa^7^.

The study herein seeks to address the role of pepsin at various pHs in a co-culture model featuring neutrophils and airway epithelial barriers^9–14^. Evidence of pepsin exposure leading to airway epithelial damage, barrier leakiness, and/or neutrophil breach of the epithelium would suggest a potential for the capacity of pepsin to exert a pathological response, if present in the lungs. Additionally, gastric fluid was collected from children who were either taking or not taking PPIs and evaluated in the co-culture model to determine whether gastric fluid was capable of triggering airway responses and whether any such response was driven by pepsin and/or impacted by the PPI status of the donor.

## Results

**Figure 1:**
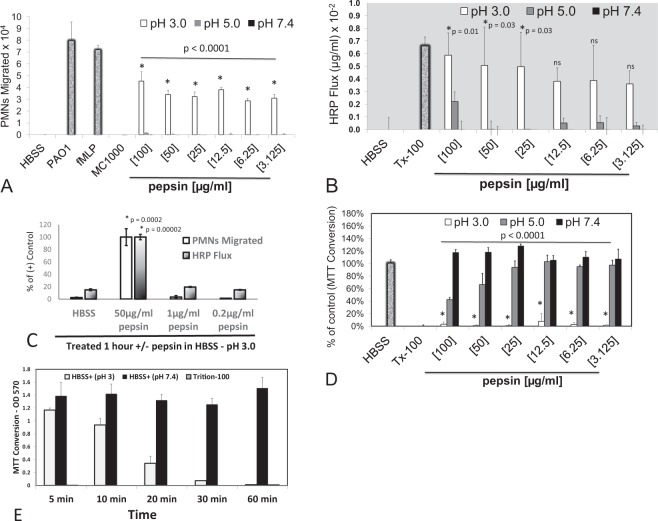
Neutrophil migration, barrier integrity disruption, and cytotoxicity in response to varying pepsin concentrations and pH. The impact of pepsin on H292 monolayers following a 1-hour exposure was examined at a concentration range of 0.2 to 100 µg/ml, suspended in HBSS at pH 3, 5, and 7.4. Pepsin (3 to 100 µg/ml) suspended in pH 3 only induced significant neutrophil (PMN) migration (**A**) and increased HRP flux (**B**). Pepsin (≤1 µg/ml) suspended in pH 3 failed to cause a significant PMN migratory response or increase HRP flux. (**C**) A complete reduction in MTT conversion was observed at all concentration of pepsin (3 to 100 µg/ml) suspended in pH 3 (**D**) and complete reduction in MTT conversion was observed by 1 hour of treatment in pH 3 buffer without pepsin. (**E**) All positive and negative assay controls were conducted in pH 7.4 HBSS. For neutrophil migration, HBSS alone and infection with non-pathogen *E. coli* (MC1000) served as negative controls and a gradient of 100 nM fMLP and infection with pathogenic *P. aeruginosa* (PAO1) served as positive controls. For HRP flux and MTT conversion, HBSS alone served as a negative control and treatment with 0.1% detergent triton X-100 (Tx-100) served as a positive control. Panels A–E are representative internally controlled experiments conducted on at least three separate occasions yielding similar results. Each data point depicts the mean +/− SD of quadruplicate wells assayed (n = 4). Differences between pepsin (pH 3) and HBSS (pH 7.4) negative control were considered significant at p ≤ 0.05 and noted by the symbol (*). P values involving multiple comparison were calculated by one way ANOVA with Dunnett’s test to compare the impact of individual pepsin concentrations (at pH 3) with HBSS control (at pH 7.4) within an internally controlled experiment.

To determine whether pepsin is capable of instigating inflammatory events, a human co-culture model of neutrophil trans-epithelial migration was evaluated in the context of pepsin exposure^11,12^. Pepsin was applied to epithelial cells at a range of pH to reflect the variability of pH found in gastric fluid. Consistent with previous studies^15^, neutrophils migrated across lung epithelial barriers in response to an imposed chemotactic gradient of fMLP or in response to apical infection with *Pseudomonas aeruginosa* (strain PAO1) (Fig. 1A). Neutrophils added to lung epithelial cultures incubated in HBSS buffer alone or infected with non-pathogenic *E. coli* (strain MC1000) do not migrate across the epithelial barrier (Fig. 1A). Pepsin, suspended in HBSS at either pH 5 or 7.4 and applied to the apical surface of H292 monolayers for 1 hour, did not elicit significant neutrophil trans-epithelial migration within a treatment dose range of 3–100 µg/ml (Fig. 1A). However, pepsin suspended in HBSS at pH 3 triggered significant neutrophil trans-epithelial migration at all doses examined between 3 and 100 µg/ml (Fig. 1A).

Since pepsin is a protease that works efficiently at low pH, it was next determined whether pepsin was capable of disrupting H292 airway epithelial barrier integrity in the absence of neutrophils. To assess barrier integrity, a horse radish peroxidase (HRP) flux assay was employed following H292 monolayer treatment with HBSS (pH 7.4), triton X-100 (Tx-100), and various concentrations of pepsin suspended in HBSS (pH 3, 5, or 7.4). After exposure to stimuli for 1 hour, the paracellular probe HRP was added to the basolateral side and the amount of HRP that transferred across the epithelial barrier to the apical side over the course of 2 hours was quantified. Buffer alone elicited minimal HRP flux, whereas treatment with Tx-100, a detergent that disrupts the epithelial barrier, caused substantial HRP flux (Fig. 1B). Pepsin applied to the airway epithelial surface for 1 hour did not result in increased HRP flux when pepsin was suspended in pH 7.4 HBSS (Fig. 1B). A similar case was observed with pepsin suspended in pH 5 HBSS, albeit trending slightly towards increased flux of HRP, particularly at 100 µg/ml (Fig. 1B). In contrast, increased HRP flux was observed at all concentrations of pepsin (3–100 µg/ml) tested when suspended in pH 3 HBSS and this increase reached statistical significance at exposure concentrations ranging from 25–100 µg/ml (Fig. 1B). In contrast to higher concentrations of pepsin in pH 3 HBSS (50 µg/ml), pepsin suspended in pH 3 HBSS at concentrations of 1 µg/ml and below failed to instigate neutrophil transmigration and did not cause increased HRP flux when were applied to lung epithelial cells (Fig. 1C).

Lung epithelial cells exposed to pepsin suspended in pH 3 buffer, as described in the above experiments, are likely rendered non-viable because of cellular exposure to extremely low pH for 1 hour. Consistent with this hypothesis, MTT conversion was not readily detected within H292 monolayers pre-exposed to pH 3 HBSS irrespective of pepsin concentration (Fig. 1D). MTT conversion within H292 monolayers was not diminished by pepsin at pH 7.4 as the magnitude of enzymatic conversion compared to H292 monolayers exposed to HBSS pH 7.4 alone with or without pepsin was not significantly different (Fig. 1D). A similar case was observed with pepsin suspended in pH 5 HBSS, although a trend towards decreased MTT conversion was observed at the higher doses of 100 µg/ml and 50 µg/ml. Treatment of H292 monolayers with Tx-100 did not exhibit any MTT conversion, indicating non-viable H292 monolayers (Fig. 1D).

**Figure 2:**
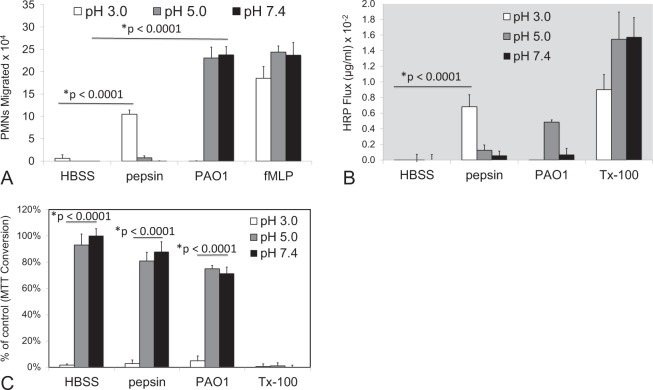
Neutrophil migration, barrier integrity disruption, and cytotoxicity in response to pepsin compared with PAO1 infection at various pHs. Pepsin (50 µg/ml) induced significant neutrophil (PMN) migration (**A**) and significantly diminished barrier integrity (**B**) at pH 3, but not pH 5 or 7.4. PAO1, in contrast, induced significant neutrophil migration at pH 5 and 7.4, but not pH 3 (**A**) and did not impact HRP flux at pH 3 and pH 7.4 (**B**). Treatment with HBSS set at pH 3 significantly decreases MTT conversion regardless of stimuli (**C**). The chemoattractant fMLP (100 nM) represents a positive control for neutrophil migration at all pHs (A). 0.1% Triton-100 (Tx-100) represents a positive control for increased HRP flux and decreased MTT conversion at all pHs (**B**,**C**). Panels A–C are representative internally controlled experiments conducted on at least three separate occasions yielding similar results. Each data point depicts the mean +/− SD of multiple wells assayed (n = 7). Differences were considered significant at p ≤ 0.05 and noted by the symbol (*). The p values were determined using an unpaired two-tailed student’s T test with equal variance within an internally controlled experiment comparing stimuli to HBSS at a matched pH (**A**,**B**) or pH 3 to pH 7.4 for each stimuli (**C**).

Since pepsin treatment of H292 airway epithelial monolayers abrogated MTT conversion at pH 3 in a similar manner regardless of pepsin concentration, the impact of acid in the absence of pepsin was explored in several contexts. Firstly, the impact of H292 monolayer exposure to acid alone was explored and it was determined that exposure to pH 3 HBSS by itself caused complete abrogation of MTT conversion by 1 hour of exposure (Fig. 1E). Secondly, HBSS at pH 3 alone for 1 hour failed to elicit significant neutrophil trans-epithelial migration (Fig. 2A) and did not alter HRP flux (Fig. 2B), similarly to pretreatment with HBSS pH 5 and 7.4. This result is consistent with the observation that low concentrations of pepsin (0.2–1 µg/ml) suspended in HBSS pH 3 (Fig. 1C) are unable to cause neutrophil trans-epithelial migration or enhance the amount of HRP that moves across the barrier. When infecting with PAO1 suspended in HBSS of varying pHs, pH 5 and 7.4 induced robust neutrophil trans-epithelial migration whereas PAO1 in HBSS pH 3 failed to induce neutrophil trans-epithelial migration (Fig. 2A). Significant neutrophil transmigration was observed across H292 monolayers in response to an imposed fMLP chemotactic gradient regardless of the pH of a 1-hour pre-exposure to buffer alone (Fig. 2A). Consistent with Fig. 1, treatment of H292 lung epithelial monolayers with pepsin (50 µg/ml) for 1 hour, only when suspended in pH 3 HBSS, caused significant neutrophil trans-epithelial migration and resulted in diminished barrier integrity as assessed by flux of HRP across the monolayer. When measuring MTT conversion to assess cell metabolic activity, all conditions involving pre-treatment in pH 3 buffer resulted in complete loss of cellular MTT conversion (Fig. 2C). Tx-100 increased HRP flux (Fig. 2B) and rendered monolayers nonviable (Fig. 2C) irrespective of pH. Taken together, these data suggest that acid alone drives a loss of cell viability, whereas pepsin in combination with acid is necessary to trigger additional inflammatory and damage related events such as neutrophil trans-epithelial migration and increased HRP flux. To further assess the impact of pepsin in combination with acid exhibiting the full extent of cellular damage and inflammation observed in this model system, epithelial monolayers were treated for a minimum of 1 hour in all subsequent experiments.

**Figure 3:**
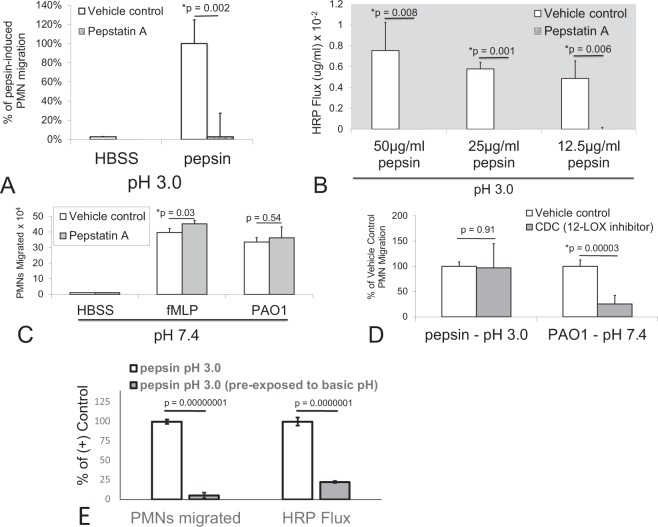
Mechanisms of pepsin-induced neutrophil migration and barrier integrity disruption. Pepstatin A (20 µg/ml), an inhibitor of pepsin, blocked neutrophil (PMN) migration induced by exposure to pepsin (50 µg/ml) at pH 3 (**A**) and prevented pepsin-induced barrier integrity disruption (HRP flux) at doses of pepsin ranging from 12.5–50 µg/ml pepsin at pH 3 (**B**). Pepstatin A failed to prevent neutrophil migration induced by PAO1 infection or following the application of a chemoattractant gradient of fMLP (100 nM) (**C**). An inhibitor of 12-lipooxygenase (CDC) failed to interfere with neutrophil migration induced by pepsin at pH 3 while PMN migration in response to PAO1 infection at pH 7.4 was significantly reduced (**D**). Exposure of pepsin (50 µg/ml) to NaOH bringing the solution pH up to between 8.5 and 10 followed by return to pH 3 by adding HCl and then applying to H292 lung epithelial monolayers abolished both the PMN transmigratory response and the increased HRP flux associated with control pepsin (50 µg/ml) at pH 3 treated in parallel with HCl only (**E**). Panels A–E are representative internally controlled experiments conducted on at least three separate occasions yielding similar results. Each data point depicts the mean +/− SD of triplicate wells assayed (n = 3). Differences were considered significant at p ≤ 0.05 and noted by the symbol (*). The p values were determined using an unpaired two-tailed student’s T test with equal variance within an internally controlled experiment.

To determine if pepsin-specific effects observed during H292 monolayer exposure in pH 3 buffer were due to the proteolytic activity associated with pepsin at low pH, we employed the pepsin activity specific inhibitor pepstatin A^16–18^. Pepstatin A (20 µg/mL) was sufficient to block pepsin-induced neutrophil migration across H292 monolayers exposed to 50 µg/ml pepsin at pH 3 (Fig. 3A). Pepstatin A also sufficiently blocked HRP flux caused by pre-exposure of H292 monolayers to pepsin at pH 3 within concentrations ranging from 12.5–50 µg/ml when the inhibitor was included with pepsin during the 1-hour incubation (Fig. 3B). Pepstatin A did not, however, have any effect on neutrophil trans-epithelial migration induced by an imposed fMLP gradient or PAO1 infection at pH 7.4 (Fig. 3C). A 12-lipoxygenase inhibitor (CDC), known to interfere with PAO1-induced neutrophil trans-epithelial migration had no effect on pepsin-induced neutrophil trans-epithelial migration (Fig. 3D), suggesting that pepsin at pH 3 triggers neutrophil trans-epithelial migration through a distinct mechanism than infection at neutral pH^15,19,20^.

To further probe the question of whether the proteolytic activity of pepsin was required to manifest these inflammatory and barrier disrupting events, a well-known feature of this digestive enzyme was exploited. The proteolytic activity of pepsin is irreversibly lost when pepsin is subjected to a basic solution of a pH higher than 8^16^. Pepsin suspended in pH 3 HBSS was exposed to NaOH, thereby raising the pH of the solution to above 8.5. After 15 minutes, the pepsin solution was adjusted back to pH 3 using HCl. Pepsin in a pH 3 HBSS solution, having been exposed to strongly basic pH, was incapable of inducing neutrophil trans-epithelial migration and failed to cause an increase in flux of HRP compared to pepsin in a pH 3 HBSS solution that had not been previously exposed to NaOH (Fig. 3E).

**Figure 4:**
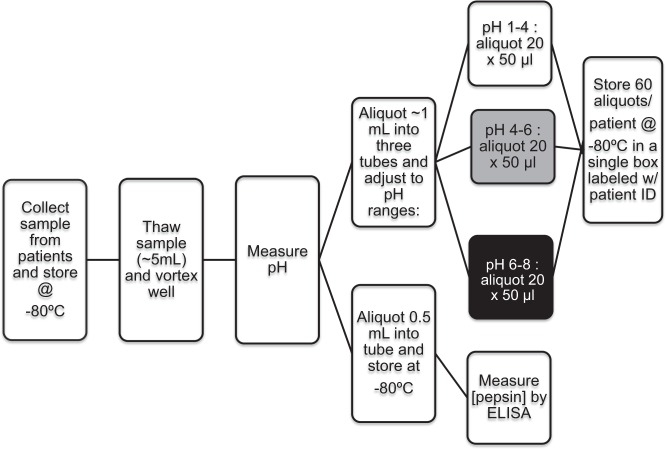
Gastric sample collection and processing. The flowchart represents the workflow following acquisition of gastric fluid samples collected from patients participating in the research study conducted at Boston Children’s Hospital.

Pepsin is one of many substances found in gastric fluid that has the potential to be refluxed and aspirated into the airway^21^. It is unclear whether individuals treated with proton pump inhibitor (PPI) therapy exhibit more pepsin or less pepsin in their gastric fluid compared to individuals not receiving therapy, or whether any such differences would influence the inflammatory triggering potential of gastric fluid when encountering airway mucosal surfaces. Gastric fluid samples from twenty-four children that were either on (n = 12) or off (n = 12) PPI therapy at the time of gastric fluid harvesting were thus collected and processed to analyze a variety of properties (Fig. 4). The average demographics of each group is listed in Table 1 and specific demographics of each individual patient is listed in Supplementary Table S1.

**Table 1:** Collective Details of Study Participants Consented to Provide Gastric Fluid Samples.

	OFF PPI therapy (n = 12)	ON PPI therapy (n = 12)	p-value^A^
Median age in years (range)	10.1 (6.3–17.2)	12.2 (3.1–19.0)	0.74
Gender	7 females, 5 males	5 females, 7 males	n/a
Average gastric fluid pH – mean (SEM)	1.44 ± 1.00	2.17 ± 1.47	0.17
Median PPI dose in mg/day in (range)	n/a	40 (15–60)	n/a

^A^The pH values from both “OFF PPI” and “ON PPI” therapy groups represent the mean +/− SEM from 12 patients/group (n = 12). Differences were considered significant between the two patient groups at p ≤ 0.05. The p values were determined using an unpaired two-tailed student’s T test with equal variance. Supplementary Fig. S1 depicts the pH of gastric fluid from each individual patient sample, with numerical values of pH listed in Supplementary Table S1 for each patient.

**Figure 5:**
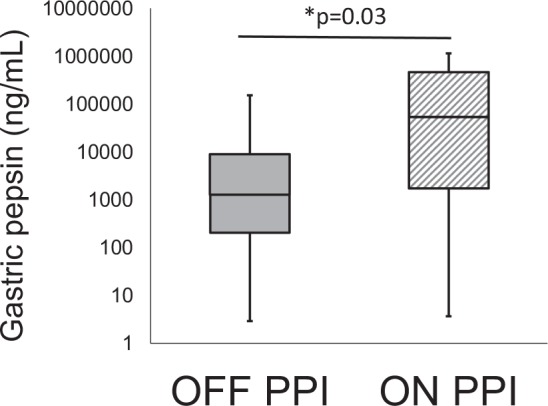
Measurement of gastric fluid pepsin concentration. Pepsin concentration was measured in gastric fluid of patients on and off PPI therapy. Gastric fluid from patients on therapy had significantly higher concentrations of pepsin than patients off therapy (p = 0.03). Both on and off PPI therapy groups represent the mean +/− SEM of pepsin measured in the gastric fluid derived from 12 patients/group (n = 12). Pepsin concentrations were log transformed and differences were considered significant between the two patient groups at p ≤ 0.05 and noted by the symbol (*). The p values were determined using an unpaired two-tailed student’s T test with equal variance. Supplementary Fig. S2 depicts pepsin concentration within the gastric fluid of each individual patient sample, with numerical values of pepsin concentration listed in Supplementary Table S1.

There were no significant differences between PPI-treated or untreated patient treatment groups in age (p = 0.75), weight (p = 0.66), or gastric pH (p = 0.17) (Table 1 and Supplementary Table S1). Individual patient gastric fluid pH measurements are depicted in Supplementary Fig. S1 and listed in Supplementary Table S1. Gastric fluid from patients “on PPI” therapy had significantly more pepsin per ml than gastric fluid from patients “off PPI” therapy (p = 0.03) (Fig. 5). Individual pepsin concentrations of each patient are depicted in Supplementary Fig. S2 and listed in Supplementary Table S1. Of note, the patients with the highest concentrations of gastric pepsin in each group (patient 2 and patient 16) also had the highest gastric pH in their respective groups (Supplementary Figs S1–S2 and Supplementary Table S1).

The question of whether gastric fluid is capable of instigating neutrophil migration across airway epithelial monolayers when applied to the apical surface has not be rigorously investigated. Given the complexity of these biological samples, we first conducted a series of screening experiments with each patient sample to determine if individual gastric fluids could trigger reproducible effects at different dilutions and when they are adjusted to three different pHs (3, 5, and 7.4). A representative experiment involving four patient gastric fluid samples demonstrates that the greatest magnitude of induced migration occurs at pH 3, with some activity occurring at pH 7.4, and very little induced migration detectable at pH 5 (Supplementary Fig. S3). Extensive screening of all 24 patient samples using this experimental design confirmed reproducibility of impact for each gastric fluid sample. HRP flux screening assays yielded similar findings, with gastric fluid at pH 3 eliciting the largest degree of barrier disruption. However, a consistent effect on HRP flux was not observed with gastric fluid at pH 5 or 7.4 (Supplementary Fig. S4). MTT activity was preserved throughout all dilutions of gastric fluid at pH 5 and 7.4. However, any sample set at pH 3 rendered the cell metabolically inactive at all dilutions (Supplementary Fig. S5).

**Figure 6:**
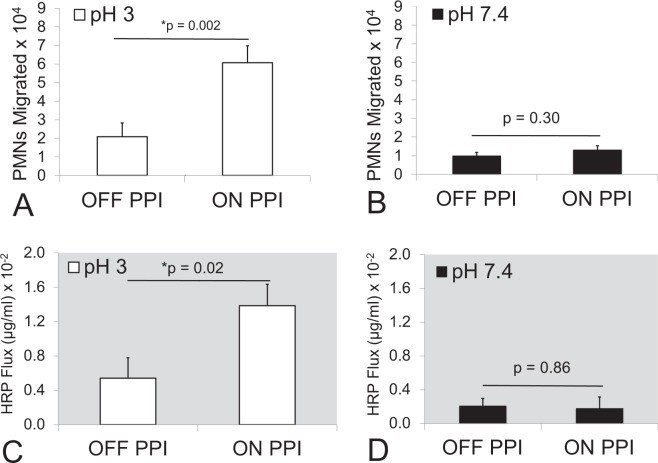
Neutrophil migration and barrier integrity disruption effects by gastric fluid set at pH 3 and pH 7.4 from patients on and off PPIs. Neutrophil (PMN) migration was significantly increased across H292 lung epithelial monolayers that had been exposed to gastric fluid from patients on PPI therapy set at pH 3 compared to monolayers that had been exposed to gastric fluid from patients off PPI therapy that were set at pH 3 (**A**). No significant differences were observed in the magnitude of neutrophil migration induced by gastric fluid derived from patients on PPI therapy and off PPI therapy set at pH 7.4 (**B**). Barrier integrity disruption as measured by HRP flux was significantly increased across lung epithelial monolayers that had been exposed to gastric fluid from patients on PPI therapy versus off PPI therapy that were set at pH 3 (**C**), whereas no difference in magnitude of HRP flux was observed between gastric fluid samples set at pH 7.4 and derived from patients on PPI therapy or off PPI therapy (**D**). A dilution of 1:4 in pH matched HBSS was used for all samples. Both on and off PPI therapy groups represent the mean +/− SEM of the average magnitude values (n = 3) of PMN migration and HRP flux induced by gastric fluid derived from 12 patients on PPI therapy and 12 patients off PPI therapy, diluted 1:4 in HBSS (n = 12). Differences were considered significant between the two patient groups at p ≤ 0.05 and noted by the symbol (*). The p values were determined using an unpaired two-tailed student’s T test with equal variance within an internally controlled experiment. Panels A–D are representative internally controlled experiments conducted on at least two separate occasions yielding similar results. Supplementary Fig. S6 depicts average magnitude of neutrophil transmigration (n = 3) and Supplementary Fig. S7 depicts average magnitude of HRP flux (n = 3) induced by gastric fluid of each individual patient sample.

Since no measurable neutrophil trans-epithelial migration response to gastric fluid was observed at pH 5, gastric fluid samples set to pH 5 were omitted from further analysis. All samples set at either pH 3 or pH 7.4 were diluted 1:4 in pH matched HBSS and examined in comparative internally controlled experiments. At pH 3, gastric fluid from patients “on PPI” therapy collectively induced significantly greater neutrophil transepithelial migration than fluid from patients “off PPI” therapy (p = 0.002) (Fig. 6A). However, this difference between groups was not observed when the gastric fluid was pH-adjusted to 7.4 and compared (p = 0.30) (Fig. 6B). Results for each individual patient gastric fluid sample are displayed in Supplementary Fig. S6A,B. When measuring HRP flux following treatment of epithelial cells with individual gastric fluid samples, a similar relationship between the “on PPI” and “off PPI” therapy groups was observed. Gastric fluid from patients “on PPI” therapy, on average, induced significantly more HRP flux at pH 3 (p = 0.02) but not at pH 7.4 (p = 0.86) (Fig. 6C,D). The results for each individual patient gastric fluid sample are displayed in Supplementary Fig. S7A,B. It is interesting to note that gastric fluid from patient 2 (off PPI) and 16 (on PPI), which were outliers in that they both measured higher pH (Supplementary Fig. S1) and had the highest pepsin concentration for their respective groups (Supplementary Fig. S2), also induced the greatest amount of neutrophil trans-epithelial migration (Supplementary Fig. S6A) and HRP flux (Supplementary Fig. S7A) when adjusted to pH 3. Furthermore, the strongest positive correlation between pepsin concentration and magnitude of induced neutrophil trans-epithelial migration was observed when plotting individual gastric fluid from patients “on PPI” therapy adjusted to pH 3 (r^2^ = 0.695) (Supplementary Fig. S6C–F).

**Figure 7:**
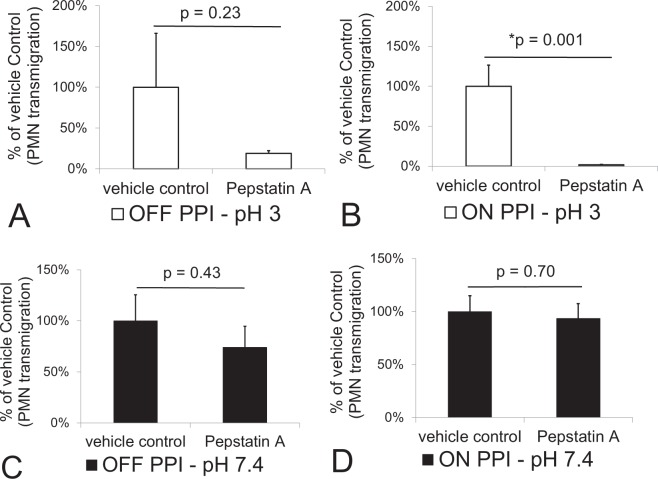
Impact of Pepstatin A on neutrophil migration induced by patient gastric fluid samples set at pH 3 and pH 7.4. Pepstatin A (20 µg/ml) was added to gastric samples set at pH 3 and pH 7.4. Pepstatin A failed to significantly reduce the magnitude of neutrophil (PMN) migration across H292 lung epithelium induced by gastric samples set at pH 3 from patients off PPI therapy (**A**), but did significantly block neutrophil migration induced by gastric samples set at pH 3 from patients on PPI therapy (**B**). For gastric fluid samples set at pH 7.4, Pepstatin A failed to impact the magnitude of neutrophil migration induced by samples collected from patients off (**C**) and on (**D**) PPI therapy. A dilution of 1:4 in pH matched HBSS was used for all samples. Both on and off PPI therapy groups set at either pH 3 or 7.4 represent the mean +/− SEM of the average magnitude values (n = 3) of PMN migration by gastric fluid derived from 12 patients on PPI therapy and 12 patients off PPI therapy, diluted 1:4 in HBSS and treated with or without Pepstatin A (n = 12). Differences were considered significant between groups with and without Pepstatin A at p ≤ 0.05 and noted by the symbol (*). The p values were determined using an unpaired two-tailed student’s T test with equal variance within an internally controlled experiment. Panels A–D are representative internally controlled experiments conducted on at least two separate occasions yielding similar results.

Next the effects of Pepstatin A on neutrophil trans-epithelial migration induced by gastric fluid samples at both pH 3 and 7.4 was examined. Pepstatin A (20 µg/ml) incubation with pH 3 gastric samples during exposure to H292 monolayers resulted in a reduction gastric sample-induced neutrophil trans-epithelial migration (Fig. 7A,B). This effect did not reach statistical significance when pH 3 gastric samples were derived from patients “off PPI” therapy (p = 0.23) (Fig. 7A) but was highly significant when pH 3 gastric samples were derived from patients “on PPI” therapy (p = 0.001) (Fig. 7B). Gastric samples at pH 7.4, on the other hand, exhibited no significant effect following addition of Pepstatin A in either the “off PPI” therapy group (p = 0.43) or the “on PPI” therapy group (p = 0.7) (Fig. 7C,D), suggesting that neutrophil migration at pH 7.4 is likely induced by factors other than pepsin.

**Figure 8:**
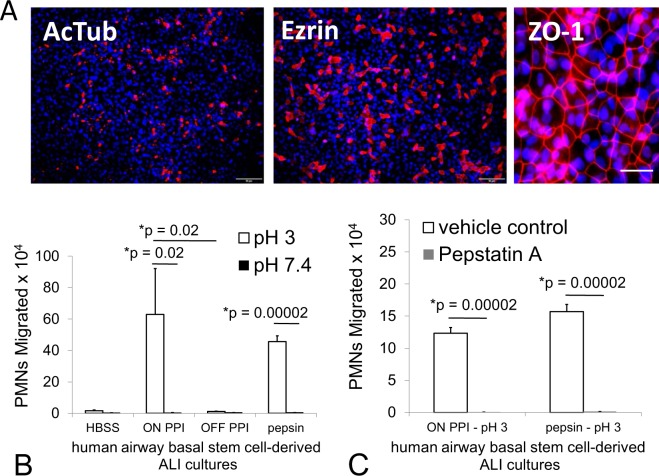
Evaluation of the impact of gastric fluid samples and pepsin on neutrophil migration using a human basal stem cell derived airway mucosa model cultured at air-liquid interface. Inverted air-liquid interface (ALI) airway mucosa cultured on inverted 3.0 μm pore sized permeable transwell filters were wholemount stained to highlight ciliated cells (AcTub & Ezrin), tight junctions (ZO-1), and nuclei (DAPI). Scale bar, 20 μm for ZO-1 field, 50 μm for AcTub & Ezrin (**A**). Pepsin (29 µg/ml) set at pH 3 and a gastric fluid sample set at pH 3 and diluted 1:4 in pH matched HBSS derived from a patient on PPI therapy both induce a significant magnitude of neutrophil (PMN) migration across a human primary airway mucosa grown at air-liquid interface (**B**). HBSS set at pH 3 and a gastric fluid sample derived from the patient off PPI therapy set at pH 3 failed to induce neutrophil migration as did all conditions set at pH 7.4 (**B**). These effects observed with pepsin and the gastric sample derived from a patient on PPI therapy, both set at pH 3, are completely abrogated by the addition of Pepstatin A (20 µg/ml) (**C**). Panels A and B are separate internally controlled experiments. Each data point depicts the mean +/− SD of triplicate wells assayed (n = 3). Differences were considered significant at p ≤ 0.05 and noted by the symbol (*). The p values were determined using an unpaired two-tailed student’s T test with equal variance within an internally controlled experiment. The gastric sample from the patient on PPI therapy was patient #18 and the gastric sample from the patient off PPI therapy was patient #11 (Supplementary Table S1).

A more physiological airway mucosal model has recently been developed and leveraged to explore mechanisms of neutrophil trans-epithelial migration^13,14^. Primary airway basal stem cells can be cultured on permeable Transwell filters at an air-liquid interface (ALI) to differentiate into a mature airway mucosa. The ALI cultures feature beating cilia, the capacity to secrete mucus, and differentiate as multiple epithelial cell types, which organize in a manner similar to the mature human lung mucosa^13,22,23^. Inverted ALIs cultured on 3.0 µm Transwell filters are a required modification to the conventional ALI Transwell model to permit the investigation of neutrophil trans-epithelial migration in a physiologically relevant direction and these cultures were fixed and stained with antibodies to detect acetylated α-tubulin (AcTub) and ezrin, which mark cilia and ciliated cells respectively (Fig. 8A). In a higher magnification image, ALI cultures are stained with antibodies to detect the tight junction protein ZO-1 to demonstrate the development of an airway mucosal barrier (Fig. 8A). The integrity of the airway mucosal barrier for all inverted ALI cultures included in this study was assessed by measurement of TEER and the measured values ranged from to 2363–13300 Ω with an average value of 8807 Ω +/− 2964. ALI cultures were treated on the apical surface with pepsin in HBSS at pH 3 and pH 7.4 for 3 hours, followed by washing and application of neutrophils to the basolateral surface. Consistent with results observed from H292 lung epithelial cell monolayers treated for 1 hour under the same conditions (Figs 1A, 2A and 3A), at pH 3, pepsin caused a substantial number of neutrophils to migrate across that was not observed in pH 3 HBSS alone or with pepsin suspended in pH 7.4 HBSS (Fig. 8B). We selected a representative gastric sample from the “off PPI” patient group (patient #11) and one from the “on PPI” group (patient #18) to examine their relative impact on inducing neutrophil trans-epithelial migration. The gastric sample adjusted to pH 3 in HBSS from the “on PPI” group induced a significant transmigratory response, whereas the pH 3 sample from the “off PPI” group did not. Neither gastric sample induced neutrophil trans-epithelial migration when the sample was adjusted to pH 7.4 in HBSS (Fig. 8B). Furthermore, addition of pepstatin A to either pepsin alone or the gastric sample from the “on PPI” group, both adjusted to pH 3, completely ablated the ability of these stimuli to induce neutrophil trans-epithelial migration (Fig. 8C). This data, examining a more physiological airway mucosal model, is consistent with our findings observed in the H292 lung monolayer model.

## Discussion

Lung dysfunction associated with extraesophageal reflux disease in children is widely reported, yet the inflammation and damage that occur within the airway resulting in the clinical presentation is not well understood. Several previous studies have explored this question by employing *in vivo* models whereby gastric fluids or components of gastric fluids are instilled into the airway of animals and the consequences to the lung are examined^24–26^. These studies have revealed that acid and other constituents of gastric fluid can cause airway inflammation and damage, with factors often exerting synergistic effects^24–26^. However, the specific mechanisms that drive the airway response to these noxious components of refluxate have remained elusive. To explore pathological mechanisms that underpin this disease state, a co-culture model of the airway mucosa and inflammatory cells was employed. The hypothesis that pepsin, a proposed biomarker of extraesophageal reflux, might also be capable of directly instigating airway inflammation and damage was tested. Pepsin triggered neutrophil migration across polarized airway epithelial barriers and disrupted epithelial barrier integrity when suspended in buffer at a low pH of 3 and applied to the apical surface of airway barriers for 1 hour. These effects were not observed when pepsin was suspended in buffer at a higher pH of 5–7.4. Treatment with buffer alone at pH 3 for 1 hour rendered airway epithelial cells metabolically inactive but did not instigate neutrophil migration across acid damaged epithelial layers, nor did low pH alone cause epithelial barrier disruption. How pepsin, in the context of an acidic environment, stimulates a process whereby neutrophils migrate across the epithelium or where degradation of epithelial integrity occurs is not entirely clear. However, proteolytic activity of pepsin appears to be required since these processes only occur with pepsin exposure in an acidic environment, where pepsin protease activity is maximal. In addition, both neutrophil transepithelial migration and barrier integrity disruption are potently blocked by the aspartyl protease inhibitor pepstatin A and when pepsin is exposed to basic pH prior to re-acidification, a maneuver that irreversibly disrupts pepsin proteolytic activity. It is also clear that the mechanism of pepsin-induced neutrophil transepithelial migration is distinct from bacterial-induced neutrophil transepithelial migration. Bacterial-induced neutrophil transepithelial migration occurs following a 1-hour infection, but only when bacteria are suspended in buffer at a higher pH (5–7.4) and does not occur when bacteria are suspended in buffer at pH 3 during infection. Further, bacterial induced neutrophil transepithelial migration is pepstatin A independent and requires epithelial 12-lipoxygenase activity^13,15,19^. The enzyme 12-lipoxygenase is likely non-functional after epithelial exposure to acidic conditions and pretreatment of the epithelium with an inhibitor of 12-lipoxygenase did not impact pepsin-induced neutrophil transepithelial migration.

Gastric fluid contains a variety of substances, all of which can be refluxed and aspirated into the lung^27,28^. Given the importance of pepsin and acid towards triggering airway inflammation (neutrophil transepithelial migration) and damage (epithelial barrier integrity disruption) observed using the human airway mucosal *in vitro* model system, gastric fluid samples were collected from children undergoing endoscopy and evaluated in the same experimental system. Since treatment with proton pump inhibitors (PPIs) is administered to raise the pH in the gastric fluid, samples were collected from patients that were on PPI therapy or off PPI therapy to determine if any difference could be detected between these two cohorts. Surprisingly, there was only a slight increase in the pH of gastric fluid samples collected from patients on PPI therapy and this mean difference did not reach statistical significance. Interestingly, there was a statistically significant increase in pepsin concentration in the patients that were on PPI therapy and this difference correlated with significant increases in the ability of gastric fluid to induce neutrophil transepithelial migration and cause airway epithelial barrier disruption. Thus, at low pHs, gastric fluid from patients on PPI therapy caused significantly more inflammation and damage when compared with gastric fluid from patients off PPI therapy. Furthermore, the acid protease inhibitor pepstatin A significantly inhibited gastric fluid-induced neutrophil transepithelial migration at low pHs when gastric fluid was derived from patients on PPI therapy, suggesting that pepsin was a major driver of inflammation within the complex milieu of the gastric fluid derived specifically from this patient cohort. Collectively, these results suggest that pepsin in combination with acid cause inflammation and damage when applied to human airway mucosal models. Furthermore, these constituents appear to be important drivers of inflammation and damage by acidic gastric fluid, particularly in patients on PPI therapy, irrespective of the potential of PPIs to raise gastric pH.

These results raise the possibility that pepsin in not just a biomarker but may have proinflammatory effects on the lung epithelium. A recent study of pepsin in tonsillar tissue reveals that pepsin can cause macrophage maturation, release of inflammatory cytokines, and triggers macrophage migration across an epithelial layer^6^. Similarly, we show that pepsin triggers neutrophil migration when in an acidic but not pH-neutral environment. Based on these results, we would expect that acid suppression would be beneficial to children with reflux related lung disease. However, when we looked at neutrophil migration across epithelium exposed to gastric fluid from PPI-treated patients, we surprisingly found that acid suppression may result in refluxate that is more destructive to the airway. Neutrophil migration was enhanced in magnitude following exposure to gastric fluid from PPI-treated patients compared with gastric fluid from untreated patients. These results raise the question that PPIs may, in fact, be more harmful than beneficial, possibly by indirectly increasing pepsin concentration in the gastric fluid as we observe herein that PPI-treated patients had higher gastric pepsin concentrations compared to untreated patients. The clinical implications of this are worth noting because PPIs are commonly used in the treatment of extraesophageal symptoms and our data suggest that while there may be a theoretical benefit to raising gastric pH, the benefit may be out weighted by the risk of increasing pepsin concentrations which may ultimately be more harmful.

While we have shown that an acidic milieu and pepsin are significant triggers of inflammation, others have shown different mechanisms by which gastric fluid may trigger inflammation^6,7,27–29^. In a study by Mertens *et al*., bronchial epithelial cells were exposed to gastric fluid from adults on and off PPIs^7^. The authors first measured the gastric pH as well as concentrations of bile, pepsin, and endotoxin and then measured IL-8 production from the bronchial epithelial cells. The authors found that bronchial epithelial cells exposed to gastric fluid with higher pHs (less acidic) produced more IL-8 than cells exposed to gastric fluid with low pH. Interestingly, the authors of this study reported a relationship between PPI status and endotoxin levels. Filtered gastric fluid was found to exhibit a significant reduction in bronchial epithelial cell IL-8 production, which the authors interpreted as due to removal of food and bacteria, potent instigators of IL-8 production. There are several reasons why our results may differ from this study. First, our gastric concentrations of pepsin were significantly higher in the PPI treated patients than those found by Mertens *et al*. suggesting why we have observed a more prominent pepsin effect on inflammation than the prior study^7^. Second, the pHs of the gastric fluid in the Mertens *et al*. study were significantly higher than those in our study. Despite PPI use, only 1/12 treated patients in our study had a pH > 4 suggesting that pepsin activation may have differed in our patients and that the likelihood of gastric bacterial overgrowth in our patients may be much less.

There are limitations to our study that merit discussion. We used both a lung epithelial model and an air-liquid interface model that were exposed to acid with or without pepsin or gastric fluid at varying pH. *In vivo*, it is reasonable to speculate that exposure to aspirated products is likely to be episodic, lower in concentration, and briefer in duration as opposed to a single, high concentration, 1-hour duration exposure used in the *in vitro* model described here. Now that we have clarified that prolonged exposure of pepsin and acid on an epithelial model together have a synergistic negative effect, the next step would be to determine if there is a threshold effect for duration or concentration that might mimic a micro-aspiration model. This process is not well understood *in vivo* and therefore it is difficult to determine the ideal *in vitro* model to mimic human disease however we suggest that this study lays the groundwork for critical future studies, particularly as we show no benefit and potential harm to acid suppression therapy. In this study, we show that an acidic environment alone had a significant impact on the viability of the model and rendered the epithelium metabolically inactive. An important next step would be to consider designing a model that would allow for much smaller acid exposure through micro-aerosolization, which may more accurately represent what the airway surface would experience. Another issue observed in this study was that the pH of gastric fluid in children taking PPIs was surprisingly low. This may be related to differences in PPI metabolism compared to adults, genetic polymorphisms, delays in PPI dosage potentially related to NPO status at the time of endoscopy, or some combination of these factors^30^. This work raises the possibility that traditional PPI regiments in children fail to prevent some degree of acid aspiration while also increasing the amount of pepsin present.

Despite these limitations, the results of this study are important because they suggest that pepsin may serve a pathological role in the lung rather than simply representing a biomarker of reflux-related lung disease. We further show that the concentration of gastric pepsin is higher in patients taking PPIs, thus raising the possibility that aspirated gastric contents in patients taking PPIs may have a greater proinflammatory and/or damaging effect and might contribute to a worsening of airway function in patients diagnosed with extraesophageal reflux.

## Methods

### Cell culture and establishment of H292 polarized lung epithelial monolayers

The human lung epithelial cell line H292 was obtained from American Type Culture Collection (Manassas, VA) and grown on the underside of 0.14 cm^2^ 96-well 3.0 µm HTS Transwell^TM^ filters (Corning, Corning, NY) as described previously^11^. Wells were coated with 30 µg/ml rat tail collagen (Invitrogen, Carlsbad, CA) prior to seeding. H292 cells were maintained in 10% FBS RPMI-1640 medium containing 1% penicillin-streptomycin and cultured at least eight days prior to use. Passages ranged from passage 4 to passage 35. Cultures are maintained with a volume of 235 µl media in the bottom chamber (apical side) and 80 µl media within the inner Transwell (basolateral side).

### Establishment of an inverted human primary airway mucosa on air-liquid interface

Air-liquid interface (ALI) differentiated human primary cell cultures were generated as a model that physiologically reflects the airway mucosa, featuring functional cilia and goblet cells capable of secreting mucus^13,22,23^. These cultures were generated from basal stem cells isolated from a single donor without lung disease as previously described^22,23^. Basal cells were cultured using a recent modification of the ALI model whereby basal cells were seeded on the underside of individual 0.33 cm^2^ inverted 3.0 µm Transwells sized for 24-well plates (Corning product #3415) and cultured for 1 to 2 days prior to establishment of air-liquid interface, whereupon media was removed from the bottom well (apical side)^13,14^. This maneuver is necessary to conduct neutrophil trans-epithelial migration experiments in a physiologically relevant manner^13,14^. ALI cultures were grown for at least 9 days to generate a functional airway barrier but no longer than 34 days to avoid overgrowth or loss of barrier function. Media (200–300 µl) was added to the inner well of the Transwell (basolateral side) and changed every other day until differentiation was well established. Once ALI cultures reached maturity, cells were examined by confocal microscopy and neutrophil trans-epithelial migration assays were conducted. A voltmeter (EVOM2) was purchased from World Precision Instruments, Inc. to assess trans-epithelial electrical resistance (TEER) in evaluation of ALI cultures for inclusion in these studies in order to confirm epithelial barrier integrity prior to conducting assays. At room temperature, ALI cultures were fixed with 4% paraformaldehyde (PFA) for 10 min, washed, and permeabilized with PBS + 0.2% Triton X-100. Then, ALIs were treated with primary antibodies against cellular markers of various states of differentiation, followed by PBS + 1% bovine serum albumin (BSA) exposure for two hours at room temperature or 4 °C overnight (>16 hrs). After incubation, ALIs were washed with PBS + 0.2% Triton X-100 for 4 rinses and placed with secondary antibodies for a room temperature incubation of 1–2 hours. After rinsing 4 times with PBS that also has 0.2% Triton X-100, cells within ALI cultures were stained with 0.1 µg/ml DAPI (4′,6-diamidino-2-phenylindole) prior to mounting slides and recording images. ALI cultures stained as describe above were visualized using the IX81 inverted fluorescence microscope from Olympus. Several planes of focus were captured and combined using Olympus Soft Imaging Solutions, MicroSuite FIVE and Extended Focal Imaging (EFI) module to create a single in-focus image of ALI cultures^22^. Antibodies used in this study include acetylated Tubulin (Sigma, Mouse monoclonal, T7451, 1:10,000), Ezrin (Developmental Studies Hybridoma Bank, Mouse monoclonal, CPTC-Ezrin-1, 1:300), ZO-1 (Life Technologies, Rabbit Polyclonal Antibody, 617300, 1:200) for primary antibodies. Life Technologies provided the secondary antibodies conjugated with Alexa Fluor 594 and conjugated antibodies were diluted 1:500.

### Neutrophil isolation

Neutrophils are polymorphonuclear cells (PMNs) that were derived from healthy adult human donors providing informed written consent prior to collection of blood. Blood was mixed with acid citrate/dextrose as an anticoagulant and centrifuged at room temperature with a speed of 1,000 x g and the break turned off to prevent agitation of separated layers. The majority of red blood cells (RBCs) were removed from separated blood layers by gravity sedimentation after addition of 2% gelatin. Prior to sedimentation, however, plasma and the thin white cell layer containing monocytes/lymphocytes were carefully removed by aspiration. Residual RBCs were then removed by lysis in cold NH_4_Cl lysis buffer. This technique allows for rapid isolation of functionally active PMNs (>98%) at 90% purity^11^.

### Bacterial strains

Non-pathogenic *Escherichia coli* (MC1000) and pathogenic *Pseudomonas aeruginosa* (PAO1) were grown aerobically in 3 mL of Luria-Bertani broth at 37 °C overnight^15^. Prior to treatment of airway epithelial cells, 1 mL of the bacterial cultures were centrifuged for 5 minutes at 15,800 × g and the supernatant discarded. The pellet was washed with 1 mL of HBSS and centrifuged again. The supernatant was discarded, and the pellet was brought to a final volume of 500 µl and 600 µl for MC1000 and PAO1, respectively. For infection of airway epithelial cells, this concentrated solution of bacterial cultures was diluted 1:100 in HBSS and 25 µl of the 1:100 dilution was used to infect H292 monolayers cultured on 96-well 3.0 µm HTS Transwell^TM^ filters and human primary airway basal stem cell derived ALI cultures^11–14^.

### Human purified pepsin

Human gastric fluid was collected from patients attending the laryngology outpatient clinic at Froedtert Hospital, Milwaukee, WI, scheduled to have a transnasal esophagoscopy for clinical indications. Approximately 5 to 10 mL of gastric fluid was aspirated from patients at the end of their scheduled procedure. Gastric fluid samples were pooled and pepsin 3b isolated by anion exchange chromatography. Gastric fluid was initially filtered through a piece of 113 V, 320-mm diameter Whatman filter paper (Fisher Scientific, Pittsburgh, PA) to remove mucoid and particulate matter. The filtrate was then dialyzed against 50 mmol/L sodium acetate buffer, pH 4.1 at 4 °C. Partial purification of pepsins was performed using a diethyl amino ethyl cellulose column (DE52, 2.5 × 10 cm, Whatman, Inc., NJ) equilibrated with dialysis buffer at 4 °C. Negatively charged proteins, including pepsin 3b, were eluted from the DE52 column with salt (sodium acetate/1 mol/L NaCl at 2 mL/min). Fractions containing protein (determined by measuring optical density at 280 nm) were pooled and dialyzed against 50 mmol/L sodium acetate at 4 °C to remove all salt before performing the second high-resolution anion exchange chromatography with a Pharmacia Mono Q, 0.5 × 5 cm, column (GE Health Care, Piscataway, NJ) developed with a linear gradient of 0.09 to 1.0 mol/L NaCl in 50 mmol/L sodium acetate, pH 4.1, over 30 minutes at 0.75 mL/minute. Pepsin 3b was identified by chromatography pattern, isolated, and stored at −20 °C in 50% glycerol (Supplementary Fig. S8)^31,32^.

### Human gastric fluid samples

Gastric fluid was collected from patients undergoing GI endoscopy (EGD) for evaluation of cough, esophageal esophagitis (EoE), gastroesophageal reflux disease (GERD), Celiac disease, or abdominal pain at Boston Children’s Hospital. Patients were included in the “on” treatment group if they had been taking PPIs for a minimum of three months and included in the “off” treatment group if they had been acid suppression naive or had been off PPIs for a minimum of three months (An exception to this was one patient who had been off PPIs for only three days) (Supplementary Table S1). Patients who had gastrostomy tubes, nasogastric tubes, taken antibiotics in the past month, or had a history of gastrointestinal surgery were excluded.

Gastric fluid samples were collected under anesthesia during EGD by suctioning into a sterile leukitrap. A minimum of 3 ml of gastric fluid was then aliquoted into a sterile 5 ml micro-centrifuge tube and kept on ice until frozen at −80 °C. Gastric fluid samples were frozen within 30 minutes of collection. To measure pH, gastric fluid was thawed on ice and mixed thoroughly by vortex. The pH was measured using the Orion STAR A211 benchtop pH meter (Thermo Scientific, Waltham, MA), which was calibrated with calibration buffer on ice. The gastric fluid was then aliquoted into three separate 1.5 ml sterile micro-centrifuge tubes and the pH was adjusted to approximately pH 3, 5, or pH 7.4 using 1N HCl or 1N NaOH. Sterile filtered HBSS at pH 3, 5, or 7.4 was used to correct for the dilution factor. Each pH range of a gastric fluid sample from a single patient was diluted to the same degree. Not more than 15% of the original volume was added to adjust pH of samples. Gastric fluid from each pH range was aliquoted into 50 µl aliquots to prevent multiple freeze-thaw cycles and stored at −80 °C (Fig. 4).

### Treatment of H292 monolayers or ALI cultures with gastric fluid or purified pepsin

Gastric fluid aliquots at each pH range were thawed on ice and diluted with filtered HBSS at the matching pH. When screening gastric fluid samples, dilutions of 1:2, 1:8, and 1:32 were used at each pH range to determine if there was a dose-dependent effect. Each condition was run in duplicate. In experiments comparing gastric fluid samples from the 12 patients off PPI therapy versus the 12 patients on PPI therapy within an internally controlled experiment, a single dilution (1:4) was selected and each sample was run in triplicate. All experiments were performed with positive and negative controls. Purified pepsin was diluted to a physiologically relevant concentration of 50 µg/ml^33^. Pepsin was diluted with purified HBSS and the pH was confirmed by measurement of solution using the Orion STAR A211 benchtop pH meter. Any adjustments to pH were made by adding small volumes of 1N HCl or 1N NaOH followed by confirmation with pH meter. In certain experiments, pepsin in solution was brought to a pH of between 8.5 and 10, before being returned to pH 3 to determine the impact of exposure to high pH on pepsin-induced effects^16^.

H292 monolayers and ALI cultures were washed with warm HBSS and allowed to equilibrate in HBSS at 37 °C, 5% CO_2_ for thirty minutes. H292 monolayers and ALI cultures were then inverted into a large petri dish and the apical side was treated with 25 µl of sample at the appropriate pH and allowed to incubate at 37 °C, 5% CO_2_ for one hour (H292 monolayer) or 3 hours (ALI cultures). After treatment, the cells were washed with warm HBSS twice before being placed in a receiver plate containing HBSS at pH 7.4. Either horseradish peroxidase (HRP) or isolated PMNs are then added to the basolateral side as described below.

### Treatment of airway cells with Pepstatin A

Pepstatin A (Sigma-Aldrich, St. Louis, MO) was resuspended in DMSO at 10 mg/ml, aliquoted, and stored at −20 °C. Aliquots were diluted to a working stock of 1 mg/mL with an addition of filtered HBSS at the designated experimental pH of either 3 or 7.4. A final concentration of 0.2% DMSO +/− 20 µg/ml pepstatin A was established in all conditions by diluting (1:50) either the 1 mg/ml pepstatin A stock solution at the appropriate pH or 10% DMSO vehicle control solution at the appropriate pH directly into gastric fluid samples, pepsin, or PAO1 prior to treating H292 monolayers or ALI cultures. All final solutions being exposed to airway cells were evaluated using the Orion STAR A211 benchtop pH meter to verify pH prior to initiating treatment.

### Neutrophil transmigration assays using H292 monolayers on 96-well transwells

After infection/treatment and washing with HBSS as described above, cells were placed into a receiver plate with HBSS pH 7.4 in the apical well. Wells containing 100 nM fMLP chemoattractant (Sigma-Aldrich, St. Louis, M.O.) served as a positive control to confirm that neutrophils were active and capable of migrating. PMNs (2 × 10^5^) in a volume of 40 µl HBSS were added to the basolateral side of each well and allowed to migrate for two hours at 37 °C, 5% CO_2_. Four blanks containing HBSS only in the absence of PMNs were included in each experiment. After incubation with PMNs for 2 hours, 96-well Transwell^™^ filter plates containing H292 monolayers were discarded and 12.5 µl of 10% Triton X-100 was added to each well in the 96-well receiver plate (containing transmigrated PMNs). A parallel plate containing a serial 2-fold dilution beginning at 2 × 10^5^ PMNs also received 12.5 µl of 10% Triton X-100/well. PMNs from a single donor were used for each internally controlled experiment. PMN transmigration results are represented as number of PMNs that traversed the monolayer, calculated from a standard PMN dilution curve. Receiver plates were incubated at 4 °C for 20 minutes, while shaking, to fully lyse PMNs in detergent. A volume of 12.5 µl citrate buffer was then added to each well at room temperature. Finally, 250 µl ABTS (2,2′-azino-bis(3-ethylbenzothiazoline-6-sulphonic acid) peroxidase substrate solution (Sigma-Aldrich, St. Louis, MO) was added to each well and allowed to develop at room temperature for 10 minutes, protected from light. Following incubation, optical density was determined at 405 nm. The number of PMNs that crossed the H292 monolayer in each sample was quantified by the myeloperoxidase (MPO) activity. The standard curve of known numbers of PMNs was used to convert OD@405 measurements of unknown wells to number of PMNs migrated^11,12^.

### Neutrophil transmigration assays using ALI cultures

After infection/treatment and washing with HBSS as described above, ALI cultures were then placed in wells containing HBSS. PMNs (1 × 10^6^) in a volume of 200 µl were added to the basolateral side of each well and allowed to migrate for two hours at 37 °C, 5% CO_2_. After incubation for 2 hours with PMNs, ALI cultures were discarded and migrated neutrophils were quantified similarly as described above with slight modifications to the volumes of Triton X-100, citrate buffer, and ABTS added as detailed previously^13,14^.

### Measurement of HRP flux across H292 monolayers grown on 96-well transwells

After 1 hour infection/treatment, H292 monolayers on 96-well Transwells were washed as described above and placed into a receiver plate containing HBSS. A concentration of 0.5 µg/ml horseradish peroxidase (HRP) (Sigma-Aldrich, St. Louis, M.O.) in a volume of 40 µl of was added to the basolateral side of each well and incubated for 2 hours at 37 °C, 5% CO_2_. Four blanks containing HBSS in the absence of HRP were included in each experiment as well as a standard curve comprised of 2-fold serial dilutions of HRP beginning at 0.5 µg/ml. After incubation for 2 hours, the magnitude of HRP flux was determined by sampling 10 µl from each well on the apical side and transferring to a separate 96-well plate. A volume of 200 µl ABTS peroxidase substrate solution was added and incubated for 10 minutes at room temperature, protected from light. After incubation, optical density was measured at 405 nm. The amount of HRP that crossed the monolayer in each sample was reported as the magnitude of HRP flux (µg/ml) using a standard curve of known HRP concentration and measurements at OD@405^12^.

### Assessment of MTT conversion within H292 monolayers grown on 96-well transwells

After measurement of HRP flux, all remaining supernatant was discarded and 96-well Transwell^TM^ filters with polarized H292 monolayers were washed with HBSS and transferred to a new 96-well receiver plate. Receiver plates contained 115 µl HBSS and 55 µl of Component A (MTT) from the Vybrant® MTT Cell Proliferation Assay Kit (Invitrogen, Carlsbad, CA)^34^. Two blanks containing no cells were included in a separate 96-well plate and both plates were incubated overnight at 37 °C, 5% CO_2_. The MTT Assay involves the conversion of the water soluble MTT (3-(4,5-dimethylthiazol-2-yl)-2,5-diphenyltetrazolium bromide) to an insoluble formazan. After overnight incubation, the formazan was then solubilized by the addition of 50 µl Component B (SDS) and incubated at 37 °C, 5% CO_2_ for four hours. Following incubation, 60 µl of the solubilized formazan in each well was mixed thoroughly by pipetting up and down and transferred into the corresponding empty wells in the plate already containing the two blanks. A volume of approximately 140 µl–160 µl was removed from the blank wells to equal the volume of the samples. Optical density was read at 570 nm.

### Assessment of pepsin concentrations in human gastric fluid samples by ELISA

The concentration of pepsin in specimens was determined by noncompetitive indirect sandwich ELISA as previously described with minor modifications^32^. Briefly, Nunc-Immuno Maxisorp™ 96-well flat bottom microtiter plates (Thermo Scientific, Waltham, MA) were coated with affinity purified rabbit anti-Hu3 antibody [3 μg/mL], produced against the N-terminus of mature human pepsin, in a volume of 100 μL 0.2 M sodium carbonate buffer, pH 9.6, per well, and incubated at room temperature for 18–20 hours. Plates were washed three times for two minutes per wash in phosphate buffered saline, pH 7.4 with 0.1% Tween-20 (PBS-T) and incubated with blocking buffer (SuperBlock™ PBS Blocking Buffer; Thermo Scientific, Waltham, MA) for 90 minutes at 37 °C and 200 rpm on an orbital shaker (Orbit, Lab-line Incorporated, Melrose, IL). Plates were rinsed three times for two minutes per wash in phosphate buffered saline, pH 7.4 (PBS). Gastric specimens and pepsin standards (purified human pepsin 3b isolated from human gastric juice by ion exchange chromatography by Mark Lively, Wake Forest School of Medicine) were prepared in sample diluent [1% bovine serum albumin (BSA; Sigma-Aldrich, Carlsbad, CA) in PBS]. All gastric specimens were diluted at least 1:5 in sample diluent. Sample diluent alone was assayed in duplicate as a blank. After specimens and standards were applied to the microtiter plate in duplicate in a volume of 100 μL per well, the plate was incubated for one hour at 37 °C. Samples were removed from the plate by vacuum aspiration and wells were washed three times for two minutes per wash in PBS-T. Captured pepsin was detected by incubation with goat anti-human pepsin antibody diluted to 26 µg/ml in 1% BSA/PBS-T for 40 minutes at 37 °C and 200 rpm. The plate was washed three times for two minutes per wash in PBS-T and incubated with peroxidase-conjugated mouse anti-goat immunoglobulin G (Sigma-Aldrich) diluted 1:30,000 in 1% BSA/PBS-T. Enzymatic color development was carried out using 1-Step Ultra TMB ELISA (Sigma-Aldrich). Color development was stopped by the addition of an equal volume of 1N sulfuric acid and the wells read at 450 nm in the microplate reader (SpectraMax Plus, Molecular Devices, Sunnyvale, CA). Duplicate readings for each standard, specimen and the blank were averaged and the average absorbance of the blank specimen was subtracted from average absorbance of specimens and standards. A standard curve was plotted using blank-subtracted mean absorbance and concentrations of pepsin standard dilutions. The concentration of pepsin in gastric specimens was calculated using a best fit curve model.

### Statistical analyses

All experiments were conducted on at least two separate occasions yielding similar results. Experiments from internally controlled experiments comparing mean of average values from clinical samples (12/group) derived from the “on” PPI therapy group versus “off” PPI therapy group are displayed as mean +/− SEM. Each data point in all other experiments represents the mean +/− SD. Differences were considered significant at p ≤ 0.05 using, in most cases, an unpaired two-tailed student’s T test with equal variance within an internally controlled experiment. Where different, specific statistical tests are described within the figure legends of each individual experiment.

### Study approval

Four separate human studies protocols were approved by institutional review boards prior to collecting human samples for the investigations described herein. The protocol permitting human gastric fluid collection from patients for the isolation and purification of pepsin was reviewed and approved by the Institutional Review Board at the Medical College of Wisconsin, study # PRO 00004759. Human airway basal stem cells were isolated from discarded human airways harvested from the New England Organ Bank or Massachusetts General Hospital (MGH) Department of Pathology following approval of IRB protocol #2010P001354 by MGH. IRB Protocol #06-10-0439 was approved by Boston Children’s Hospital to obtain gastric fluid samples from children either taking or not taking proton pump inhibitors. Protocol #1999P007782 was approved by MGH to obtain blood from healthy adult volunteers to isolate neutrophils. Written informed consent was received from all participants and/or from parent/guardians, when applicable, prior to inclusion in each of these human study protocols. All methods were carried out in accordance with the relevant guidelines and regulations.

## Supplementary information

LaTeX Supplementary File

## Data Availability

All data generated or analyzed during this study are included in this published article (and its Supplementary Information files).
