# Supplementary material for: Pepsin Triggers Neutrophil Migration Across Acid Damaged Lung Epithelium
Source: Sci Rep. 2019 Sep 24;9:13778. doi: 10.1038/s41598-019-50360-4 (PMC6760148; doi:10.1038/s41598-019-50360-4)
Supplement: Supplementary file 1 — LaTeX Supplementary File [file 41598_2019_50360_MOESM1_ESM.docx]

**Pepsin Triggers Neutrophil Migration Across Acid Damaged Lung Epithelium**

**Supplementary Data**

Bryan P. Hurley^1^**^*^**, Rebecca H. Jugo^1,2^, Ryan F. Snow^1^, Tina L. Samuels^3^, Lael M. Yonker^1^, Hongmei Mou^1^, Nikki Johnston^3^, Rachel Rosen^2*^

^1^Department of Pediatrics, Mucosal Immunology & Biology Research Center,

Massachusetts General Hospital, Harvard Medical School, Boston, Massachusetts

^2^Aerodigestive Center, Department of Gastroenterology and Nutrition, Boston’s Children’s Hospital, Harvard Medical School, Boston, Massachusetts

^3^Departments of Otolaryngology and Communication Sciences, and Microbiology and Immunology, Medical College of Wisconsin, Milwaukee, Wisconsin

**^*^**Corresponding authors:

Bryan Hurley, Ph.D. Rachel Rosen, M.D., M.P.H.

Mucosal Immunology & Biology Research Center Division of Gastroenterology & Nutrition

Massachusetts General Hospital Boston’s Children’s Hospital

55 Fruit Street, Jackson 1402 300 Longwood Ave

Boston, MA 02114 Boston, MA 02115

Office: (617) 726-3101 Office: (617) 355-6055

[bphurley@mgh.harvard.edu](mailto:bphurley@mgh.harvard.edu) [Rachel.Rosen@childrens.harvard.edu](mailto:Rachel.Rosen@childrens.harvard.edu)

**Supplementary Results Figure Legends:**

**Supplemental Figure S1: Gastric fluid pH measurements from individual patient gastric fluid samples.** No significant differences were found between samples from the on PPI (n=12) and off PPI (n=12) therapy groups (p=0.17). Mean +/- SEM of each group is listed in Table 1 and the statistical analysis comparing these two groups is described within a footnote of Table 1. Individual pH values for each patient gastric fluid sample that correspond to this bar graph are listed in Supplemental Table 1.

**Supplemental Figure S2: Measurement of the concentration of pepsin within individual patient gastric fluid samples**. The pepsin concentration of each individual patient gastric fluid sample was measured by ELISA. Samples from patients on PPI therapy (n=12) collectively had a significantly greater concentration of gastric pepsin than samples from patients off PPI therapy (n=12) (p=0.03). Mean +/- SEM of each group is depicted in Figure 5 and the statistical analysis comparing these two groups is described in the Figure legend for Figure 5. Individual pepsin concentration values for each patient gastric fluid sample that correspond to this bar graph are listed in Supplemental Table 1.

**Supplemental Figure S3: Screening gastric samples in a neutrophil migration assay.** All 24 gastric fluid samples used in this study, 12 from patients on PPI therapy and 12 from patients off PPI therapy, were set at pH (3, 5, and 7.4) and pre-screened in groups of 4 samples to measure the magnitude of neutrophil migration (OD@405nm) across lung epithelial monolayers following exposure to dilutions of (1:2, 1:8, and 1:32) in pH matched HBSS. This figure depicts a representative experiment of 4 gastric fluid samples screened in parallel. A greater magnitude of neutrophil migration across lung epithelial monolayers was seen in response to gastric samples set at pH 3, followed by pH 7.4, with minimal responses elicited by samples set at pH 5. Controls and all samples at all dilutions were examined in duplicate on at least two separate occasions yielding similar results. “s” is an abbreviation for sample #. Assay controls were all set at pH 7.4. Negative controls included HBSS alone and infection with non-pathogenic *E. coli* (MC1000). Positive controls included infection with pathogenic *P. aeruginosa* (PAO1) as well as establishment of a 100 nM gradient of fMLP.

**Supplemental Figure S4: Screening gastric samples in a barrier integrity disruption assay.** All 24 gastric fluid samples used in this study, 12 from patients on PPI therapy and 12 from patients off PPI therapy, were set at pH (3, 5, and 7.4) and pre-screened in groups of 4 samples to measure the magnitude of disruption of lung epithelial monolayer barrier integrity following exposure to dilutions of (1:2, 1:8, and 1:32) in pH matched HBSS. This figure depicts a representative experiment of 4 gastric fluid samples screened in parallel. A greater magnitude of HRP flux (OD@405nm) across lung epithelial monolayers was seen in response to gastric samples set at pH 3, followed by pH 7.4, with minimal disruption elicited by samples set at pH 5. Controls and all samples at all dilutions were examined in duplicate on at least two separate occasions yielding similar results. “s” is an abbreviation for sample #. Assay controls were all set at pH 7.4. Negative controls included HBSS and infection with P. aeruginosa (PAO1). Positive controls included treatment with 0.1% triton-100 (Tx-100) as well treatment with trypsin-EDTA.

**Supplemental Figure S5: Screening gastric samples in cytotoxicity assay.** All 24 gastric fluid samples used in this study, 12 from patients on PPI therapy and 12 from patients off PPI therapy, were set at pH (3, 5, and 7.4) and pre-screened in groups of 4 samples to measure cytotoxic effects on lung epithelial monolayers following exposure to dilutions of (1:2, 1:8, and 1:32) in pH matched HBSS. This figure depicts a representative experiment of 4 gastric fluid samples screened in parallel. MTT conversion (OD@570nm), a measurement of viable cell metabolic activity, was decreased in all samples set at pH 3, with minimal cytotoxic effects observed at pH 5 or 7.4. Controls and all samples at all dilutions were examined in duplicate on at least two separate occasions yielding similar results. “s” is an abbreviation for sample #. Assay controls were all set at pH 7.4. Negative controls included HBSS and infection with P. aeruginosa (PAO1). The positive control was treatment with 0.1% triton-100 (Tx-100).

**Supplemental Figure S6:** **Neutrophil migration induced by gastric fluid set at pH 3 and pH 7.4 from patients on and off PPI therapy**. Neutrophil (PMN) migration across lung epithelial monolayers elicited by each individual patient gastric fluid sample was measured for samples set to pH 3 (A) and pH 7.4 (B). Samples set at pH 3 from patients on PPI therapy (n=12) collectively induced a significantly greater magnitude of neutrophil migration than samples set at pH 3 from patients off PPI therapy (n=12) (p=0.002) (A). No significant difference was observed between on and off PPI therapy groups with samples set at pH 7.4. Mean and standard deviation of each group is depicted in Figure 6A and 6B and the statistical analysis comparing these two groups is described in the Figure 6 legend. Assay controls were all set at pH 7.4. Negative controls included HBSS alone and infection with non-pathogenic *E. coli* (MC1000) (A & B) as well as pepsin (B). Positive controls included infection with pathogenic *P. aeruginosa* (PAO1) (A & B) as well as establishment of a 100 nM gradient of fMLP (A). Correlation curves comparing pepsin concentration to the magnitude of the PMN trans-epithelial migratory response are depicted for gastric fluid samples from patients off PPI therapy set at pH 3 (C), from patients on PPI therapy set at pH 3 (D), from patients off PPI therapy set at pH 7.4 (E), and from patients on PPI therapy set at pH 7.4 (F).

**Supplemental Figure S7:** **Barrier integrity disruption elicited by gastric fluid set at pH 3 and pH 7.4 from patients on and off PPIs**. Barrier integrity disruption of lung epithelial monolayers elicited by each individual patient gastric fluid sample was measured for samples set to pH 3 (A) and pH 7.4 (B). Samples set at pH 3 from patients on PPI therapy (n=12) collectively induced a significantly greater disruption of barrier integrity as measured by HRP flux than samples set at pH 3 from patients off PPI therapy (n=12) (p=0.02) (A). No significant difference was observed between on and off PPI therapy groups with samples set at pH 7.4. Mean and standard deviation of each group is depicted in Figure 6C and 6D and the statistical analysis comparing these two groups is described in the Figure 6 legend. Negative controls included HBSS alone set at pH 7.4 and infection with *P. aeruginosa* (PAO1) in pH 7.4 HBSS (A & B) Positive controls included treatment with 0.1% triton-100 (Tx-100) (A & B) as well as treatment with pepsin set at pH 3 (A) and treatment with trypsin-EDTA set at pH 7.4 (B).

**Supplemental Figure S8: Purification of human pepsin from gastric juice**. Human pepsin from gastric juice (Medical College of Wisconsin Institutional Review Board Protocol Number PRO00004759) was purified by column chromatography using a diethylaminoethyl (DEAE) column to concentrate the pepsin, followed by separation on a MonoQ anion exchange column and high-performance liquid chromatography (HPLC). Separation of pepsin 3b from pepsin 3c and pepsin 3a (all 35KDa) and other minor proteins on the MonoQ column was accomplished using NaCl gradients in the primary buffer (ammonium acetate, 50 mM, pH 4.1). Fractions containing protein were identified using absorbance readings at 280 nm. The pepsin containing fractions were identified by comparing the migration of sample on a 12.5% sodium dodecyl sulphate polyacrylamide gel electrophoresis (SDS-PAGE) to that of known protein molecular weight markers. Pepsin 3b fractions were pooled, dialyzed to remove salts, and then concentrated by centrifuging the sample through a molecular weight cutoff filter of 6,000-8,000 Daltons. The final pepsin product was assayed for activity against a protein peptide substrate (Lys-Pro-Ala-Glu-Phe-PNP-Arg-Leu-COOH, molecular weight = 1,052.18; PNP = paranitrophenylalanine) This peptide is cleaved specifically by pepsin between Phe-5 and PNP-6. Cleavage was monitored by measuring the decrease in A300 as the pepsin degraded the substrate. Identification of protein containing fractions from human gastric juice (A), MonoQ column purification of pepsin (B), 12.5% SDS-PAGE gel for pepsin 3b, (C), and Activity assay for pepsin 3b (D).

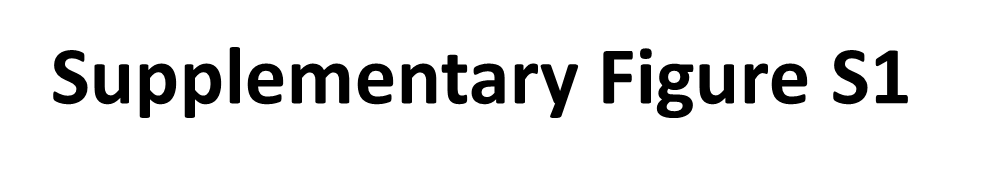

**Supplementary Figure S2**

**Supplementary Figure S3**

**Supplementary Figure S4**

**Supplementary Figure S5**

**Supplementary Figure S6**

**Supplementary Figure S7**

**B**

**A**

**Supplementary Figure S8**

**D**

**C**
